# The Actin Cytoskeleton Responds to Inflammatory Cues and Alters Macrophage Activation

**DOI:** 10.3390/cells11111806

**Published:** 2022-05-31

**Authors:** Elsa Ronzier, Alexander J. Laurenson, Rohini Manickam, Sophia Liu, Imelda M. Saintilma, Dillon C. Schrock, John A. Hammer, Jeremy D. Rotty

**Affiliations:** 1 Department of Biochemistry and Molecular Biology, Uniformed Services University of the Health Sciences, Bethesda, MD 20814, USA; elsa.ronzier.ctr@usuhs.edu (E.R.); alex.laurenson@gmail.com (A.J.L.); rohini.manickam@usuhs.edu (R.M.); sophia.liu.ctr@usuhs.edu (S.L.); meredith.imelda@gmail.com (I.M.S.); 2 The Henry M. Jackson Foundation for the Advancement of Military Medicine, Bethesda, MD 20817, USA; 3 Cell and Developmental Biology Center, National Heart, Lung, and Blood Institute, National Institutes of Health, Bethesda, MD 20892, USA; dillon.schrock@nih.gov (D.C.S.); hammerj@nhlbi.nih.gov (J.A.H.)

**Keywords:** actin cytoskeleton, Arp2/3 complex, myosin-II, macrophages, iNOS, inflammation, CCL22

## Abstract

Much remains to be learned about the molecular mechanisms underlying a class of human disorders called actinopathies. These genetic disorders are characterized by loss-of-function mutations in actin-associated proteins that affect immune cells, leading to human immunopathology. However, much remains to be learned about how cytoskeletal dysregulation promotes immunological dysfunction. The current study reveals that the macrophage actin cytoskeleton responds to LPS/IFNγ stimulation in a biphasic manner that involves cellular contraction followed by cellular spreading. Myosin II inhibition by blebbistatin blocks the initial contraction phase and lowers iNOS protein levels and nitric oxide secretion. Conversely, conditional deletion of Arp2/3 complex in macrophages attenuates spreading and increases nitric oxide secretion. However, iNOS transcription is not altered by loss of myosin II or Arp2/3 function, suggesting post-transcriptional regulation of iNOS by the cytoskeleton. Consistent with this idea, proteasome inhibition reverses the effects of blebbistatin and rescues iNOS protein levels. Arp2/3-deficient macrophages demonstrate two additional phenotypes: defective MHCII surface localization, and depressed secretion of the T cell chemokine CCL22. These data suggest that interplay between myosin II and Arp2/3 influences macrophage activity, and potentially impacts adaptive-innate immune coordination. Disrupting this balance could have detrimental impacts, particularly in the context of Arp2/3-associated actinopathies.

## 1. Introduction

The actomyosin cytoskeleton contains actin filaments (F-actin), actin binding proteins (ABP) and non-muscle myosin that together maintain cell shape, drive cell movement and control cellular mechanosensing [1,2,3]. Indeed, tight spatiotemporal regulation of actin assembly and filament arrangement allows cells to maintain and regulate diverse cellular functions that occur simultaneously: membrane protrusion, endocytosis and exocytosis, vesicle trafficking, organelle positioning and phagocytosis. Dynamic cytoskeletal rearrangements also play crucial roles in higher order cellular processes such as gene transcription, cellular signaling and cell–cell communication [4,5,6].

Actin filaments are assembled from monomeric globular actin (G-actin) at the barbed end (+) of the nascent filament [5]. Though several actin assembly factors can generate linear actin filaments, there is only one known mechanism for generating the branched networks that feature in many dynamic actin-dependent cellular functions: the Actin Related-Protein 2/3 complex (Arp2/3 complex). The Arp2/3 complex is composed of seven subunits ARP2, ARP3, and ARPC1-5 [7,8,9,10]. The ARPC2 and ARPC4 subunits form a functional dimer and have been described as the primary F-actin side-binding interface [7,11]. ARP2 and ARP3 form templates for the polymerization of a nascent ‘daughter filament’ at a characteristic ≈70° branch angle and, like actin itself, these subunits hydrolyze ATP [12]. 

By virtue of its role as a major actin nucleator, many cellular functions have been attributed to Arp2/3 [4]. Recent work using Arp2/3 complex knockdown or knockout strategies confirmed that Arp2/3 complex is involved in lamellipodia formation, membrane ruffling and cell migration, echoing previous work in the field [13,14,15,16,17]. In addition to its role in cellular protrusion, Wu and colleagues found that Arp2/3-deficient fibroblasts up-regulate cytokine secretion due to enhanced NF-kB activation [18]. Thus, Arp2/3 depletion is sufficient to induce inflammation in fibroblasts, with surprising non-autonomous effects on surrounding cells [18]. This suggests that the actomyosin cytoskeleton may regulate MAPK signaling, though there are likely additional signaling pathways that are sensitive to loss of the Arp2/3 complex and branched actin, or sensitive to alterations in the actin cortex. As key inflammatory effectors, crosstalk of MAPK and NF-kB with the actin cytoskeleton may be relevant in physiological responses. Disruption of several Arp2/3-activating proteins correlates with heightened inflammation. For example, a restricted deletion of the *Rac1* gene in mouse keratinocytes leads to a stronger inflammatory response compared to WT mice [19]. Rac2, cdc42, WIP, DOCK8 and DOCK2 mutations have all been implicated in autoimmune and autoinflammatory disorders affecting humans [20,21,22,23,24,25,26]. 

All of the aforementioned findings point to a class of human disorders termed ‘actinopathies’. Perhaps the best known member of this class of maladies is Wiskott–Aldrich Syndrome (WAS), in which patients present with elements of immunodeficiency and autoinflammation [27]. The causative mutations are in the Arp2/3-activating Nucleation Promoting Factor (NPF) WASP [28]. In vitro assays demonstrate profound Arp2/3 deficiencies in WAS patient-derived leukocytes [29]. WASP-deficient cells were recently discovered to hyper-activate the Type I IFN response, due to endosomal structural defects that aberrantly activate cGAS/STING [30,31]. Recent work has also identified patient mutations in the Hem1 subunit of the WAVE regulatory complex (another NPF) that present with autoimmunity and persistent viral infections [32]. Mutations in the Arp2/3 complex itself have also been implicated in human disease. Kahr and colleagues found that the loss of the ARPC1B subunit causes platelet abnormalities and immunodeficiency and also predisposes to inflammatory disease [33]. Though much has been learned from these and related studies, it is possible that additional molecular mechanisms exist that link the actin cytoskeleton to immunoregulation.

Here, we demonstrate that the macrophage actomyosin cytoskeleton strikes a temporal balance between Arp2/3 and myosin II activities as it responds to inflammatory co-stimulation with LPS and Interferon gamma (IFNγ). Macrophages initially become contractile and reorganize actomyosin structures around the nucleus, which is reversed during continued stimulation, as cells spread out and demonstrate less association between actin and myosin. Blocking the spreading response with Arp2/3 deletion increases inducible nitric oxide synthase (iNOS) activity. Conversely, disrupting myosin II contractility with blebbistatin blocks the early contractile response and decreases both iNOS protein and its activity. The actomyosin cytoskeleton appears to be acting post-transcriptionally on iNOS regulation, possibly by altering its susceptibility to proteasomal degradation. In addition to altering iNOS activity, Arp2/3 complex deletion suppresses MHCII surface localization and depresses secretion of the Treg stimulating chemokine CCL22. These data indicate that the actomyosin cytoskeleton responds to inflammatory stimuli and is capable of influencing macrophage function. Altering actomyosin regulation in macrophages may therefore affect the innate immune system’s ability to communicate efficiently with the adaptive immune system. This lack of coordination between the adaptive and innate immune responses may contribute to elements of actinopathy-induced immune dysregulation. Findings presented here also support the notion that disrupting the actin cytoskeleton may have non-autonomous consequences.

## 2. Results

### 2.1. The Actin Cytoskeleton Responds to Inflammatory Stimulation in Two Temporal Phases

**Figure 1:**
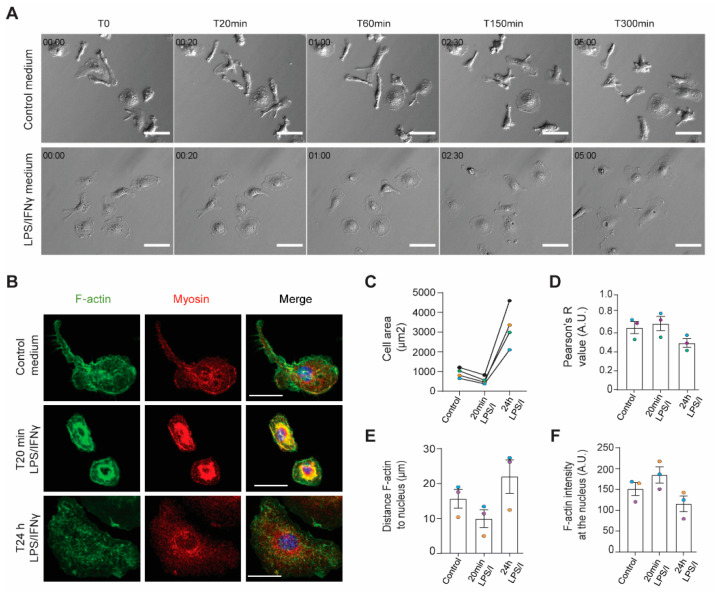
The actin cytoskeleton responds to inflammatory stimulation in two temporal phases. (**A**) Relief contrast images of macrophages at 0 min, 20 min, 60 min, 150 min and 300 min (5 h) after LPS/IFNγ or control medium wash-in. Scale bar = 50 µm. (**B**) Representative confocal z-stack images of myosin IIA and phalloidin staining in WT macrophages during standard culture conditions, or after 20 min or 24 h of LPS/IFNγ stimulation. Scale bar = 20 µm. (**C**) Average cell area in µm^2^ of WT macrophages at different time points after LPS/IFNγ stimulation. Phalloidin staining of confocal images was used as a mask to define cell edges and measure cell area. Time points within each experimental replicate are linked. N = 4 independent experiments, with at least 14 cells analyzed per condition in each experiment. (**D**) Average Pearson’s R value used to describe overlap between myosin IIA and phalloidin in confocal images in WT macrophages during indicated conditions. N = 3 independent experiments, with at least 14 cells analyzed per condition in each experiment with means color coded for individual experiments. Error bars represent standard error of the mean. (**E**) Average distance along a line trace in microns between F-actin maximum intensity and the nucleus. N = 3 independent experiments, with at least 14 cells analyzed per condition in each experiment with individual experimental means color-coded across conditions. Error bars represent standard error of the mean. (**F**) Average F-actin intensity at the nucleus, as measured from line traces across the cell. N = 3 independent experiments, with at least 14 cells analyzed per condition in each experiment with individual experimental means color-coded across conditions. Error bars represent standard error of the mean.

To study dynamic changes in response to inflammatory activation, we treated mouse bone marrow derived WT macrophages with Lipopolysaccharide (LPS) and Interferon gamma (IFNγ) and imaged them for 5 h. We were surprised to see an initial transient contraction in these cells (e.g., 20 min after stimulation), followed by delayed re-spreading (e.g., 300 min after stimulation) (Figure 1A, lower, represents still frames from Movie S1). The contractile phase occurred during roughly the first two hours of stimulation, with progressive spreading happening from two hours onward. Macrophages treated with fresh media were used as controls and did not demonstrate the same biphasic response (Figure 1A, upper, represents still frames from Movie S2). Macrophages fixed at distinct time points after stimulation and stained with phalloidin revealed that the acute contractile phase (e.g., 20 min) was relatively short and did not significantly alter F-actin levels, though the distribution of actin filaments in the cell was altered, becoming more perinuclear, and acutely activated macrophages were smaller than control cells (Figure 1B,C), though their height remained unchanged (Figure S1). Conversely, the spreading phase was much longer, and actin filaments were more evenly distributed across the cell than at the early time point (Figure 1B). After 24 h, these cells were much larger than at baseline (Figure 1C). These data led us to hypothesize that the biphasic response to inflammatory stimulation is driven by dynamic reorganization of actin and myosin. Co-localization of myosin IIA with F-actin is quite obvious 20 min after stimulation, especially in the perinuclear area, where F-actin is enriched during the contractile phase (Figure 1B,D–F). Live imaging of mouse bone marrow derived GFP-myosin IIA knock-in macrophages corroborated the dynamic nature of actomyosin reorganization during the contractile phase as GFP-myosin IIA, like phalloidin in fixed samples, accumulates at the nucleus and the leading edge of LPS/IFNγ-treated cells (Figure S2A–D). Tight association of myosin IIA with F-actin was released 24 h after stimulation with LPS/IFNγ, as noted by less myosin IIA-phalloidin colocalization during the spreading phase (Figure 1D). In addition, F-actin distribution is more uniform during this phase and no longer localizes strongly to the perinuclear area (Figure 1E,F). Like F-actin, myosin IIA localization was more uniformly cytoplasmic during the spreading phase (Figure 1B). Together, these data argue that the cytoskeletal response to inflammatory activation occurs in distinct temporal stages that potentially influence immune effector function.

**Figure 2:**
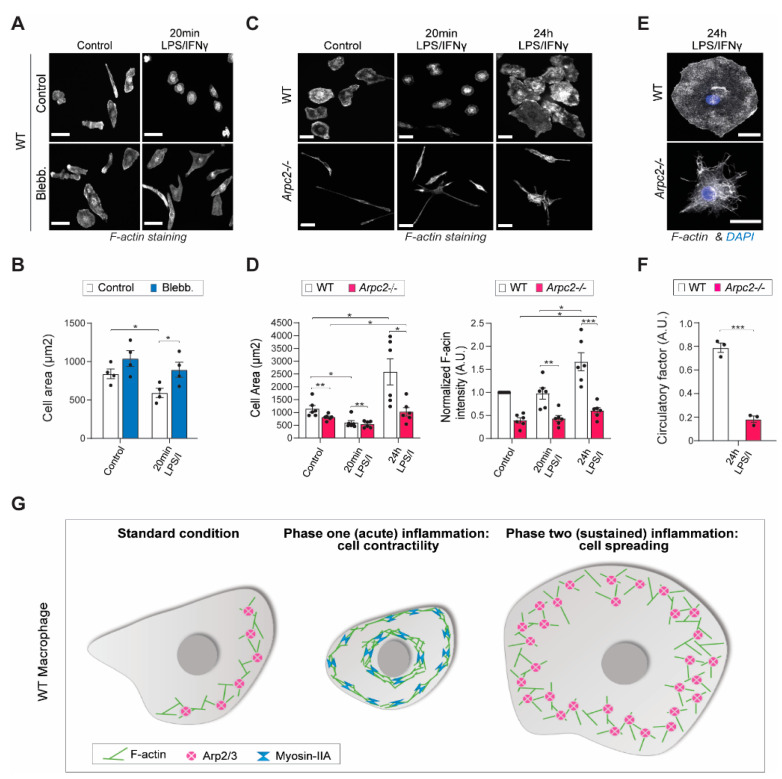
Myosin II and Arp2/3 complex reciprocally regulate cell shape changes during inflammatory activation. (**A**) Representative epifluorescence images of phalloidin staining in PFA-fixed WT macrophages pre-treated with DMSO or 30 µM blebbistatin (control) or treated with either 20 min of DMSO + LPS/IFNγ or 30 µM Blebb + LPS/IFNγ. Scale bar = 30 µm. (**B**) Average cell size (in square microns) for indicated conditions, using phalloidin epifluorescence staining as a mask for cell area. N = 3 means from independent experiments with at least 31 cells analyzed per condition in each experiment. Error bars represent standard error of the mean. *p* values were obtained with an unpaired *t*-test. * *p*-value ≤ 0.05. (**C**) Representative epifluorescence images of phalloidin staining in PFA-fixed WT and *Arpc2-/-* macrophages after 20 min or 24 h of stimulation with LPS/IFNγ. Scale bar = 30 µm. (**D**) Left, average cell size (in square microns) for indicated conditions, using phalloidin epifluorescence staining as a mask for cell area. N = 6 independent experiments with at least 8 cells analyzed per condition in each experiment. * *p* ≤ 0.05, *p* values were obtained with an unpaired t-test. Right, average of normalized F-actin intensity of WT and *Arpc2-/-* macrophages at different time points, N = 6 independent experiments, * *p* ≤ 0.05, ** *p* ≤ 0.01, *** *p* ≤0.001, *p* values were obtained with an unpaired t-test. Error bars for each measurement represent standard error of the mean. (**E**) Representative confocal images of PFA-fixed WT and *Arpc2-/-* macrophages after 24 h of stimulation with LPS/IFNγ. Scale bar = 20 µm (**F**) Average circularity factor obtained with ImageJ from WT and *Arpc2-/-* macrophages after 24 h stimulation with LPS/IFNγ. N = 3 independent experiments with at least 44 cells analyzed per condition in each experiment. Error bars represent standard error of the mean. *** *p* ≤ 0.001 (**G**) Schematic representation of cell shape changes that occur during macrophage inflammatory activation. At baseline, macrophages are well spread and have a dominant, Arp2/3 complex containing cell edge, balanced by myosin II function elsewhere in the cell. During the contractile phase, this balance between Arp2/3 and myosin II is shifted in myosin’s favor, leading to contraction. Then, as cells adjust to the inflammatory stimulus, the balance is shifted in Arp2/3′s favor, leading to spreading.

We next wanted to determine whether the two phases were controlled by myosin II contractility and Arp2/3 complex-mediated spreading. Indeed, macrophages exposed to the myosin II inhibitor blebbistatin do not contract significantly after 20 min of LPS/IFNγ stimulation compared to both unstimulated, blebbistatin-treated cells and unstimulated, DMSO-treated control cells (Figure 2A, B). We generated Arp2/3 complex KO (*Arpc2-/-*) macrophages [16] (Figure S3A–C) and found that loss of Arp2/3 complex significantly impaired cell spreading during the second phase of LPS/IFNγ response (Figure 2C,D). Though impaired, the *Arpc2-/-* macrophages retained a minimal ability to spread (Figure 2C,D), indicating that linear actin filaments may also contribute to the spreading response. In addition, loss of Arp2/3 complex impaired F-actin generation both at baseline and in response to LPS/IFNγ in *Arpc2-/-* macrophages (Figure 2D). Loss of Arp2/3 also impacted cell shape. *Arpc2-/-* macrophages lacked the characteristic circular appearance of their WT counterparts 24 h post-stimulation (Figure 2E,F), providing evidence that inflammation-induced cell shape changes are impaired without the Arp2/3 complex. Taken together, these experiments reveal that the actomyosin cytoskeleton responds in a biphasic fashion to inflammatory stimulation and that the two distinct phases are reciprocally regulated by myosin II and Arp2/3 complex activity that together influence cell size and shape, as well as F-actin levels and organization (Figure 2G).

### 2.2. Arp2/3 Complex and Myosin II Reciprocally Regulate iNOS Activity

**Figure 3:**
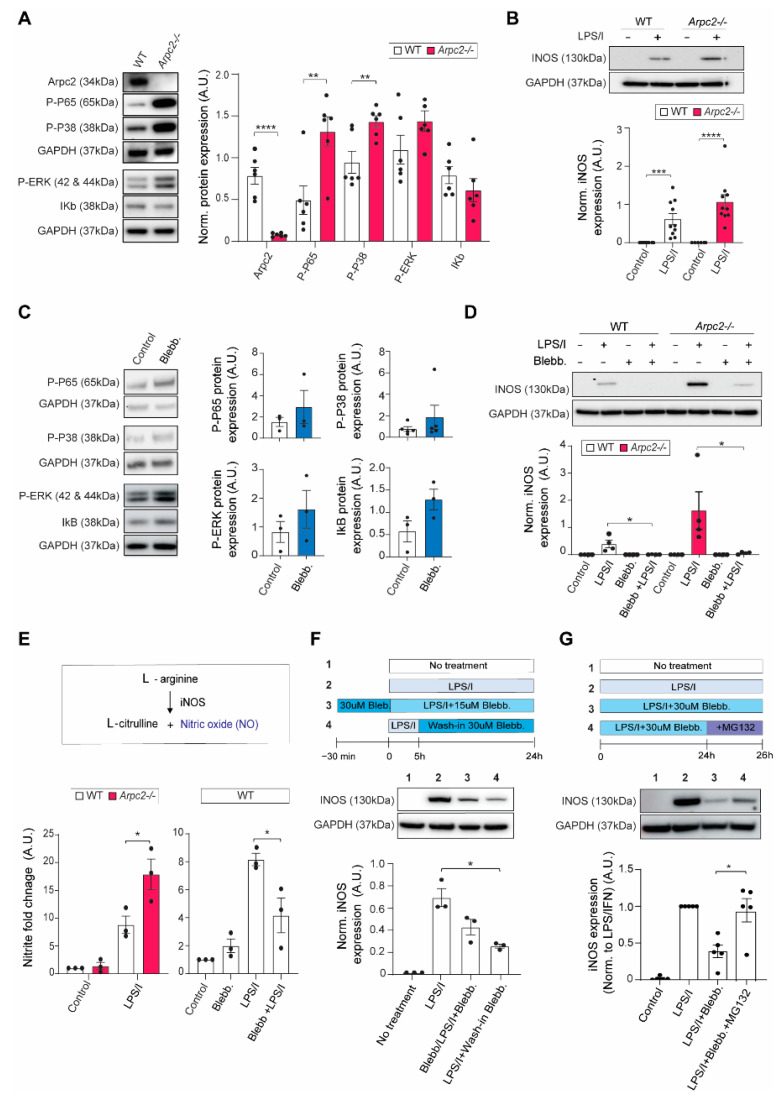
Arp2/3 complex and myosin II differentially regulate iNOS levels and activity. (**A**) Left, representative immunoblot of Arpc2, p-p65 (NF-kB), p-p38 (MAPK), p-Erk1/2, IkB and GAPDH, from unstimulated WT and *Arpc2-/-* RIPA-extracted protein lysates. Right, average chemiluminescence level (normalized to GAPDH) from N = 6 independent experiments. Error bars represent standard error of the mean. * *p* ≤ 0.05, ** *p* ≤ 0.01, **** *p* ≤0.0001, *p*-values obtained with an unpaired *t*-test. (**B**) Top, representative immunoblot for iNOS and GAPDH from WT and *Arpc2-/-* macrophages under control conditions (normal media) or after exposure to 500 ng/mL LPS + 25 pg/mL IFNγ. Right, average of iNOS levels (normalized to GAPDH) from N = 10 independent experiments. Error bars represent standard error of the mean. *** *p* ≤ 0.001, **** *p* ≤ 0.0001. (**C**) Top, representative immunoblots of p-p65 (NF-kB), p-p38 (MAPK), p-Erk1/2 and IkB, from unstimulated control (DMSO-treated) and unstimulated + blebbistatin treated WT macrophage RIPA-extracted protein lysates. Bottom, average protein expression level (normalized to GAPDH) from N = 3–5 independent experiments, as indicated on each graph. Error bars represent standard error of the mean. (**D**) Top, representative immunoblot for iNOS and GAPDH from WT and *Arpc2-/-* macrophages pre-treated with DMSO or 30 µM blebbistatin followed by 24 h treatment with DMSO + LPS/IFNγ or 15 µM blebbistatin + LPS/IFNγ. Bottom, average iNOS protein expression (normalized to GAPDH), N = 3 independent experiments, *p*-values were obtained with an unpaired t-test. Error bars represent standard error of the mean. * *p*-value ≤ 0.05. (**E**) Top, schematic of nitric oxide production via L-arginine oxidation catalyzed by iNOS. Bottom left, nitrite fold change normalized by cell number for WT and *Arpc2-/-* macrophages in standard culture conditions or after 24 h of LPS/IFNγ stimulation. N = 3 independent experiments. * *p* ≤ 0.05, as measured by unpaired t-test. Bottom right, nitrite fold change in WT DMSO or WT blebbistatin-treated macrophages ±24 h of LPS/IFNγ stimulation. DMSO or blebbistatin were pre-treated for 3 h prior to inflammatory stimulation. N = 3 independent experiments. Error bars represent standard error of the mean. * *p* ≤ 0.05, as measured by unpaired *t*-test. (**F**) Top, schematic of experimental design. WT macrophages were unstimulated (untreated), stimulated for 24 h with LPS/IFNγ, stimulated for 24 h with LPS/IFNγ + blebbistatin (including a short pre-treatment with blebbistatin), or stimulated for 5 h with LPS/IFNγ followed by blebbistatin wash-in and continuance in LPS/IFNγ media. Middle, representative immunoblot for iNOS and GAPDH from each of the conditions outlined above. Proteins were extracted in RIPA buffer. Bottom, average iNOS protein expression (normalized to GAPDH), N = 3 independent experiments. Error bars represent standard error of the mean. *p*-values were obtained with an unpaired *t*-test. * *p*-value ≤ 0.05. (**G**) Top, schematic of experimental design. WT macrophages were unstimulated (untreated), or treated with LPS/IFNγ, LPS/IFNγ + blebbistatin, or LPS/IFNγ + blebbistatin combined with a 2 h wash-in of the proteasome inhibitor MG132. All samples were harvested at 26 h. Middle, representative immunoblots for iNOS and GAPDH from each of the conditions outlined above. Proteins were extracted in RIPA buffer. Bottom, average iNOS protein expression (normalized to GAPDH) and expressed as fold change relative to the LPS/IFNγ treated samples, N = 5 independent experiments, *p*-values were obtained with an unpaired *t*-test. Error bars represent standard error of the mean. * *p*-value ≤ 0.05.

The actomyosin cytoskeleton responds to inflammatory stimulation, so we next wondered whether the cytoskeleton itself alters macrophage activation. Previous data have linked the Arp2/3 complex to MAPK and NF-kB regulation [18]. Consistent with this finding, the mitogen-activated protein kinase pathway (e.g., p-ERK, p-p38) and NF-kB pathway (e.g., p-p65) are stimulated in *Arpc2-/-* bone marrow derived mouse macrophages under normal culture conditions (Figure 3A). Interestingly, total levels of p65, p38 and Erk1/2 remain unchanged by Arp2/3 loss (Figure S4A). While these data suggest a baseline activation in the NF-kB pathway, IkB levels are less affected and NF-kB nuclear translocation does not occur spontaneously in unstimulated *Arpc2-/-* macrophages (Figure 3A and Figure S5A,B). Arp2/3 disruption is also not sufficient to induce pro-inflammatory cytokine transcripts in the absence of an inflammatory stimulus (Figure S5C). However, the baseline activation of MAPK/NF-kB in the *Arpc2-/-* macrophages led us to hypothesize that loss of Arp2/3 complex function could prime inflammatory stimulation. We began by analyzing a major hallmark of inflammatory activation: iNOS expression. Contrary to our expectations, loss of Arp2/3 complex function did not significantly alter iNOS protein level (Figure 3B). Pro-inflammatory cytokines were also not induced at a higher level in LPS/IFNγ stimulated, Arp2/3-deficient macrophages compared to control (Figure S5C).

Contraction dictates cell shape during the acute response to LPS/IFNγ, so we wondered whether inflammatory activation was similarly influenced by myosin II activity. Blebbistatin treatment alone did not significantly alter MAPK or NF-kB phosphorylation, nor did it influence IkB levels, or total levels of MAPKs or p65 (Figure 3C and Figure S4B). Nor was myosin II inhibition alone sufficient to transcriptionally induce pro-inflammatory cytokines or iNOS in the absence of an inflammatory stimulus (Figure S5D). However, myosin II inhibition decreased iNOS protein levels after LPS/IFNγ stimulation (Figure 3D). Blebbistatin treatment also suppressed iNOS in *Arpc2-/-* macrophages (Figure 3D). The actomyosin cytoskeleton elicits a biphasic, reciprocal change in cell size and actomyosin assembly in response to LPS/IFNγ. Production of nitric oxide (NO) in response to LPS/IFNγ is also reciprocally controlled. NO levels were higher in *Arpc2-/-* macrophages and lower in blebbistatin-treated macrophages after 24 h of LPS/IFNγ stimulation (Figure 3E). These findings led us to test whether myosin II and Arp2/3 complex regulate iNOS at the transcriptional level. To our surprise, neither perturbation significantly altered inflammatory induction of iNOS transcript (Figure S5C,D). iNOS is highly induced at the transcript level 5 h after LPS/IFNγ treatment (Figure S5C). We wondered whether blebbistatin wash-in at this time point, when iNOS transcript is highly expressed, would similarly downregulate iNOS at the protein level. If so, this would indicate that myosin regulates iNOS levels post-transcriptionally. Blebbistatin wash-in at 5 h post-LPS/IFNγ treatment suppressed iNOS protein levels similarly to cells that had been treated with blebbistatin prior to inflammatory stimulation (Figure 3F). These findings together with previous studies showing that iNOS is regulated by the proteasome [34] led us to suspect that iNOS proteolytic turnover is regulated by the actomyosin cytoskeleton. Wildtype macrophages were left unstimulated or treated overnight with LPS/IFNγ + DMSO, +blebbistatin, or +blebbistatin followed by a 2 h treatment with MG132 (a proteasome inhibitor) prior to harvesting. Even after this short recovery time, iNOS levels rebounded with MG132 treatment (Figure 3G). These data together suggest that actin-associated proteins can alter cellular signaling and tune the activity of key inflammation associated proteins, possibly by influencing them post-transcriptionally. It will be interesting to determine whether proteolytic turnover of other proteins is also regulated by the actin cytoskeleton.

### 2.3. Antigen Presentation and Th-Skewing Cytokine Secretion Are Altered by Loss of Arp2/3 Complex

**Figure 4:**
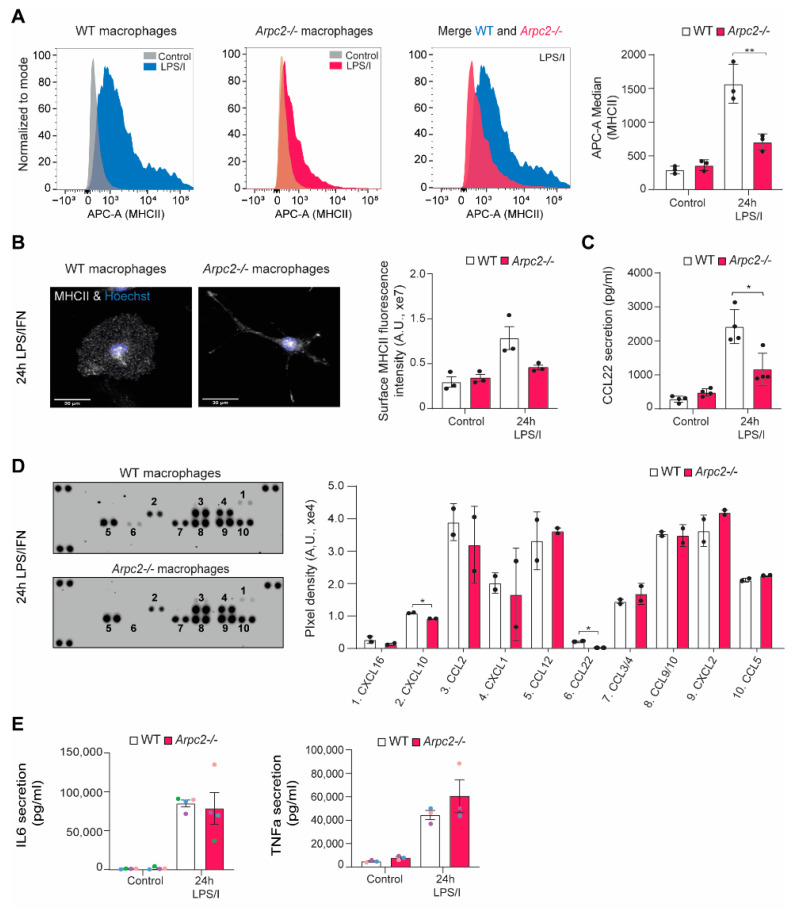
MHCII surface localization and secretion of the T_reg_ chemokine CCL22 are altered by loss of Arp2/3 complex. (**A**) Left, Representative fluorescence histograms showing MHCII surface levels (APC-A) in WT control vs. WT stimulated (LPS/I), KO control vs. KO stimulated (LPS/I) and WT stimulated versus KO stimulated (LPS/I), obtained by reading 10,000 events via FACS measurement. Plots have been normalized to the mode of the data. Right, Average median intensity of APC-A fluorescence (staining MHCII) from WT and *Arpc2-/-* macrophages left untreated or stimulated for 24 h with LPS/IFNγ, n= 3 independent experiments. ** *p*-value = 0.0089. (**B**) Left, representative images of surface MHCII staining from WT and *Arpc2-/-* macrophages at 24 h LPS/ IFNγ stimulation. Scale bar = 30 µm. Right, average cellular MHCII surface staining. Error bars represent standard error of the mean. N = 3 independent experiments with at least 29 cells analyzed per condition in each experiment. (**C**) Average CCL22 chemokine secretion by WT and *Arpc2-/-* macrophages at baseline or after 24 h of LPS/IFNγ stimulation, as measured by ELISA. N = 4 independent experiments, with *p*-values obtained via unpaired t-test. Error bars represent standard error of the mean. * *p*-value ≤ 0.05. (**D**) Left, Mouse chemokine array blot of WT and *Arpc2-/-* culture medium after 24 h LPS/IFNγ stimulation. Right, quantification of blot intensity for the 10 chemokines detected in these samples. Numbering on the graph corresponds to numbers on the raw blot data above. Each chemokine is spotted onto the membrane in duplicate. N = 2 independent experiments. Error bars represent standard error of the mean. * *p*-value ≤ 0.05. (**E**) Average IL6 and TNFα secretion by WT and *Arpc2-/-* macrophages at baseline or after 24 h of LPS/IFNγ stimulation, as measured by ELISA. Means are color coded by experiment. Error bars represent standard error of the mean. N = 4 independent experiments.

iNOS induction is a relatively late response to inflammatory activation [35]. We next sought to determine whether additional late-stage responses were altered in Arp2/3-deficient macrophages. As antigen-presenting cells, macrophages facilitate important adaptive immune functions, including T cell maturation. Innate immune cells that have been ‘matured’ with antigen or an inflammatory stimulus upregulate MHCII and Th-skewing cytokines. We hypothesized that the LPS/IFNγ-induced cell spreading noted several hours after stimulation would be critical for both antigen presentation and secretion of T cell regulating chemokines and cytokines. In line with this idea, MHCII levels at the cell surface were lower in LPS/IFNγ-stimulated *Arpc2-/-* macrophages compared to WT, as measured by both FACS and quantitative immunofluorescence (Figure 4A,B). These data indicate that the *Arpc2-/-* macrophages are likely to have an antigen presentation defect. A large number of chemokines and cytokines are secreted by LPS/IFNγ stimulated macrophages. Many of these help tune the T cell-mediated adaptive immune response. These include IL-12b, IL-23a, CCL17 and CCL22, among others. CCL22 is a T cell chemotactic cue that has been specifically implicated in Treg recruitment [36,37,38]. ELISA experiments confirmed that CCL22 secretion by LPS/IFNγ-stimulated *Arpc2-/-* macrophages is downregulated compared to their WT counterparts (Figure 4C). Chemokine array experiments confirm that CCL22 secretion is specifically downregulated by *Arpc2-/-* macrophages (Figure 4D). Out of 10 chemokines detected in these samples, only CCL22 and CXCL10 were found at lower levels in *Arpc2-/-* media after LPS/IFNγ stimulation (Figure 4D). In addition, IL-6 and TNFα are secreted and transcribed normally in both *Arpc2-/-* and blebbistatin-treated cells activated with LPS/IFNγ (Figure 4E, Figures S5C,D and S6). These data argue against a general secretory defect in Arp2/3 complex-deficient macrophages. Instead, Arp2/3 deficiency may potentially affect specific elements of macrophage effector function (NO production, antigen presentation, Th skewing) that influence the adaptive immune system.

## 3. Discussion 

We demonstrate in this study that LPS/IFN-γ stimulation induces a biphasic cytoskeletal response that influences macrophage activation. The biphasic nature of this response is due to temporal interplay dominated first by myosin II-dependent contractility followed by Arp2/3-dependent spreading. The initial contractile phase correlates with strong actomyosin association at the leading edge and in the perinuclear space. Myosin II inhibition blocked acute contraction. During the later spreading phase, actin and myosin are evenly distributed across the cell, rather than enriched at two particular locations. Genetic deletion of the Arp2/3 complex impaired the spreading phase. However, it is interesting to note that the *Arpc2-/-* macrophages still retained some spreading ability. Linear actin filaments may therefore play a role during the spreading phase of the LPS/IFNγ response, or may have a modest compensatory influence when Arp2/3 complex is compromised. *Arpc2-/-* macrophages activate MAPK/NF-kB signaling even without inflammatory stimulation, suggesting that these cells may be primed for activation. Supporting this notion, Arp2/3 complex disruption also elevates NO production in response to LPS/IFN-γ. Conversely, myosin II inhibition suppresses NO production and decreases iNOS levels. Inhibiting the proteasome rescued iNOS levels in the presence of blebbistatin, suggesting that myosin II protects iNOS from proteolytic turnover. We also discovered that *Arpc2-/-* macrophages localize less MHCII to the cell surface and secrete less of the T cell-regulating chemokine CCL22. Surprisingly, *Arpc2-/-* macrophages secrete normal levels of IL6, TNFα and numerous other chemokines, arguing against a pervasive secretory defect in Arp2/3-deficient macrophages. These findings suggest crosstalk between the actin cytoskeleton and inflammatory signaling. In total, the data presented here support the notion that the actomyosin cytoskeleton responds to inflammatory stimuli and that distinct cytoskeletal regulators can tune specific macrophage functions that may be relevant to human disease states.

One implication of this work is that a temporal balance exists between myosin IIA and the Arp2/3 complex during inflammatory stimulation. Recent work indicates that RhoA-myosin II generated intracellular pressure is sufficient to disrupt lamellipodial protrusion [39]. There is also an extensive body of literature pointing to RhoA-Rac1 antagonism [40,41,42]. These findings are in line with more recent work demonstrating that myosin II and Arp2/3 are part of a mutually antagonistic bistable system [43]. Groundbreaking work with dendritic cells (DCs) demonstrated that cell dynamics in immature DCs had two components: an Arp2/3-dependent leading edge that contributed to environmental sampling and antigen capture and a RhoA-mDia1 trailing edge that facilitated migration [44]. On the other hand, DCs that had been ‘matured’ by LPS demonstrated enhanced mDia1-mediated motility and had an Arp2/3 complex-free edge [44]. These studies and others have convincingly demonstrated that Arp2/3-myosin II interplay influences cell shape and motility. The present study suggests that inflammatory activation may be another context where this reciprocal relationship occurs. 

Similarities exist between the recently discovered Calcium-mediated Actin Reset (CaAR) system and the acute changes occurring in response to inflammation [45]. Though the temporal course of CaAR and the acute inflammatory response differ, both involve transient structural changes to the cytoskeleton that potentially drive cellular responses. Calcium influx is known to accompany inflammatory activation [46,47], which makes the possibility of a response similar to CaAR all the more compelling. Positive feedback between the stretch-activated cation-transporting Piezo1 channel and actin has been implicated in inflammatory activation [48]. Intriguingly, inhibition of myosin light chain kinase in this system inhibited Piezo-mediated calcium influx [48], suggesting that myosin-mediated contractility and traction forces play a role in calcium-dependent inflammatory activation. Along these same lines, cell shape, mechanical stretch and confinement have all been implicated as immunomodulatory factors [35,49,50,51]. Of particular interest, cell confinement (induced by impairing spreading) has been shown to have differential effects on relatively late inflammatory responses (e.g., IL6, IL1B, iNOS, CXCL9 induction) [35]. We also observe a differential effect on ‘late’ responses to inflammation (iNOS, MHCII, CCL22) upon cytoskeletal impairment. 

The actin cytoskeleton has also been implicated as a regulator of NF-kB and MAP kinases. We demonstrate that MAPK and NF-kB phosphorylation are increased at baseline in *Arpc2-/-* macrophages. Previous work has linked Arp2/3 complex to negative regulation of both MAP kinase and NF-kB in fibroblasts [18]. Though not sufficient to induce iNOS or inflammation in macrophages, loss of Arp2/3 complex can prime NF-kB/MAPK similarly to previous work done in fibroblasts [18]. In addition, the actin cytoskeleton and/or actin-associated factors have been identified as interactors of MAPK regulators, the NF-kB activator IKK and iNOS itself [18,52,53]. Consideration of MAPK and NF-kB signaling only takes the effect of LPS stimulation of TLR4 into account. Stimulation with IFN-γ activates JAK/STAT1 signaling [54]. While there is much in the literature to link MAPK and NF-kB to the actin cytoskeleton, comparatively little knowledge exists regarding actin’s potential influence on JAK/STAT signaling. However, Rac1 knockout keratinocytes demonstrate higher STAT1 phosphorylation and overall STAT1 levels [19]. Rac1 contributes to Arp2/3 complex activation, which suggests a link between branched actin polymerization and JAK/STAT signaling. Furthermore, iNOS, MHCII expression and CCL22 secretion are all regulated by IFN-γ [55,56,57]. These studies together with the present one strongly suggest crosstalk between the actomyosin cytoskeleton and IFN-γ signaling. While the present study focuses on inflammatory signaling, future studies should be conducted to determine whether the actin cytoskeleton impacts M2-like polarization (e.g., IL-4 stimulation). Given that M2-like polarization is responsive to cell morphology [49], it seems likely that differential cytoskeletal regulation will play a mechanistic role. 

The present study indicates that the actomyosin cytoskeleton post-transcriptionally regulates iNOS. iNOS is known to be post-transcriptionally regulated via Src-dependent phosphorylation [58] and MAPK-dependent Ser phosphorylation and mRNA stabilization [59,60]. iNOS is also regulated post-translationally by the proteasome [34]. iNOS is an actin binding protein [53], so myosin II-containing filaments could conceivably bind to iNOS and directly delay its proteolytic turnover. This would explain why blebbistatin treatment decreases iNOS at the protein level, without affecting its transcription. However, myosin II is also a major regulator of cortical tension. Enhanced cortical tension could suppress regulators of the ubiquitin-proteasome pathway during inflammatory activation in macrophages, leading to iNOS accumulation post-translationally. Lower cortical tension could act in a converse fashion to stimulate regulators of the ubiquitin-proteasome pathway. Changes in cortical tension might also explain how *Arpc2-/-* macrophages upregulate iNOS activity in response to LPS/IFNγ. An elegant recent study demonstrated that myosin II’s ability to penetrate the actin cortex, and thereby enhance cortical tension, is limited by its own diffusion into the cortex [61]. Myosin II penetrated farther into the cortex and enhanced cortical tension in cells treated with CK-666 (an Arp2/3 complex inhibitor), suggesting that branched actin might hinder myosin II cortical localization [61]. In light of these findings, it seems likely that *Arpc2-/-* macrophages will have higher cortical tension as well, which may alter signaling pathways that activate iNOS. 

Arp2/3 complex activation in leukocytes is multi-faceted. The WAVE regulatory complex is fundamentally linked to traveling waves that drive lamellipodial protrusion and phagocytosis [62,63,64]. WASP, on the other hand, seems to have a more localized effect, exemplified by its role in podosome function [29] and endocytosis [30,65]. WASP appears to respond to localized signals, including physical force and surface topography [62,66]. Despite these discrete contributions in some contexts, recent evidence also points to a role for both WAVE and WASP in pseudopod-based motility [67]. Given the fundamental importance of these NPFs to leukocyte function, it is not surprising that human disorders involving mutations in WASP and the WAVE regulatory complex present clinically as immunodeficiency syndromes [28,32,68]. We expected Arp2/3-deficient macrophages to present with hyper-inflammatory phenotypes compared to normal control cells; but rather than having broad defects, many elements of the inflammatory response in *Arpc2-/-* and myosin II-inhibited macrophages are functionally normal, at least with bone marrow derived mouse macrophages in vitro. Nonetheless, the phenotypes that we report here may have an outsized impact on innate-adaptive immune coordination. Antigen presentation is predicted to be severely hampered in *Arpc2-/-* macrophages, meaning that T cell expansion and maturation is likely to be affected. Previous findings in WASP-deficient dendritic cells are supportive of this idea [69]. Excessive NO produced by *Arpc2-/-* macrophages may lead to excessive tissue damage during inflammatory activation and may also improperly skew Th balance. On the other hand, decreased NO production in response to myosin II inhibition could hamper the immediate immune response, as NO is an important second messenger that can effect transcriptional changes, as well as playing a fundamental role in host defense [70,71]. Depressed secretion of T cell stimulating chemokines like CCL22 in *Arpc2-/-* macrophages could lower Treg and Th2 recruitment to inflammatory foci. Tregs directly inhibit Th1 cells and induce expression of the anti-inflammatory cytokine IL-10 by macrophages, enhancing M2-like polarization and efferocytosis [72,73,74]. Impaired Treg function is an element of autoimmunity and experimental mouse models with impaired Treg function are more sensitive to endotoxin shock [72,75]. All of these points argue that dysregulated Arp2/3–myosin balance in innate immune cells may have significant impacts on adaptive immune function. Wiskott–Aldrich syndrome, Arpc1b deficiency, Hem1 deficiency and numerous other human actinopathies present with elements of autoinflammation and immunodeficiency [32,76]. Many more cell types than macrophages are impacted by these syndromes, including platelets, neutrophils, T cells and B cells [32,76,77]. Nonetheless, the current study indicates that dysregulation of the actomyosin cytoskeleton may contribute to disease by impairing coordination between the innate and adaptive immune response. 

## 4. Materials and Methods

### 4.1. Mouse Lines Used to Derive Macrophages

WT and *Arpc2-/*- macrophages are from mice harboring a conditional *Arpc2* allele (consisting of LoxP sites flanking exon 8 of the gene encoding the p34/Arpc2 subunit of the Arp2/3 complex) that had been previously crossed to the *Ink4a/Arf−/−* and Rosa26-CreER alleles, resulting in a mixed strain background [16]. GFP-NMIIA (GFP-myosin IIA) macrophages come from homozygous GFP-tagged NMIIA knock-in mice on a C57Bl/6 background gifted by Dr. Robert Adelstein (National Heart, Lung, and Blood Institute, Bethesda, MD, USA) [78].

### 4.2. Bone Marrow-Derived Macrophage Preparation and Cell Culture

All macrophages used in this study are bone marrow derived macrophages in primary culture. Hematopoietic cells were isolated from femurs of *Arpc2* conditional or GFP-NMIIA knock-in mice. After cutting both extremities of femur using a razor blade, the bone marrow is flushed from the bone with phosphate buffered saline (PBS; Corning, Corning, NY, USA) supplemented with 2% fetal bovine serum and filtered with a 40-micron cell strainer (Falcon, Corning, Corning, NY, USA) in order to harvest and purify progenitor cells. These cells are incubated with Macrophage Media (MM) composed of DMEM (4.5 g/L D-Glucose, L-Glutamate, Sodium Pyruvate, minus phenol red (Corning, Corning, NY, USA) 10% FBS (Sigma-Aldrich, St. Louis, MO, USA) and 1% Glutamax (ThermoFisher Scientific, Waltham, MA, USA) supplemented with 30% (v/v) of L949 cell-conditioned media in a 10-cm dish (ThermoFisher Scientific, Waltham, MA, USA). Cells were maintained, for at least 7 days before experiments, on 10-cm plastic dishes in macrophage media at 37 °C, 90% humidity and 5% CO_2_. Differentiated bone marrow macrophages were sorted from the bulk population on the basis of F4/80 positivity using an Alexa 488-conjugated antibody (Biolegend, San Diego, CA, USA) and FACS-based sorting. Bone marrow-derived macrophages were then maintained in culture conditions similar to those above. When cell confluency was high (60–90%), cells were washed with PBS (Corning) and incubated with 0.5 mM cold EDTA (ThermoFisher Scientific, Waltham, MA, USA) for 10 min at 4 °C. After this incubation, EDTA was removed and cells are scraped in 1–3 mL of fresh macrophage media and re-plated at a lower density (10–30% confluency) in vessels appropriate to each experiment.

### 4.3. Antibodies

GAPDH (clone 6C5) (ThermoFisher, Waltham, MA, USA; #AM4300); Myosin IIA (Cell Signaling, Danvers, MA, USA; #3403S); iNOS (abcam, Cambridge, UK; #ab15323); Phospho-P65 NF-kB (Cell Signaling, Danvers, MA, USA; #3033S); Total p65 (Cell Signaling, Danvers, MA, USA; #8242); Phospho-p38 MAPK (Cell Signaling, Danvers, MA, USA; #9211); Total p38 MAPK (Cell Signaling, Danvers, MA, USA; #9212); Phospho-P44/P42 MAPK (ERK 1/2) (Cell Signaling, Danvers, MA, USA; #9106); Total p44/p42 MAPK (ERK 1/2) (Cell Signaling, Danvers, MA, USA; #4695); IkBa (Cell Signaling, Danvers, MA, USA; #9242); Arpc2 (Millipore, Burlington, MA, USA; #07-227); MHCII (clone M5/114.15.2) (Biolegend, San Diego, CA, USA; #107601); APC-conjugated MHCII (clone M5/114.15.2) (Biolegend, San Diego, CA, USA; #107613).

### 4.4. Cell Treatments

#### 4.4.1. Arpc2 Allele Recombination with 4-Hydroxytamoxifen (4-OHT) Treatment

BMDMs denoted as ‘WT’ in these studies carry homozygous floxed alleles of the Arpc2 gene [15,16]. To induce knockout, WT BMDMs were incubated with 2 uM of 4-OHT (Sigma-Aldrich, St. Louis, MO, USA; #H7904) in MM for 48 h, at which point media was replaced by fresh macrophage media containing 2 uM 4-OHT. On day 5, cells were split into fresh macrophage media without 4-OHT. Arpc2 KO macrophages were used in experiments 5 to 25 days after the first day of 4-OHT treatment.

#### 4.4.2. LPS and IFN-g co-Stimulation

Unless otherwise noted, macrophages were treated with Lipopolysaccharides from E. coli (LPS) (500 ng/mL, Sigma-Aldrich, St. Louis, MO, USA, #L4391) and Interferon gamma (IFNγ) (25 pg/mL, ThermoFisher Scientific, Waltham, MA, USA) in fresh macrophage media from 5 min to 24 h at 37 °C, 90% humidity and 5% CO_2_. 

#### 4.4.3. Myosin II Inhibition via Blebbistatin

Baseline blebbistatin effect on MAPK/NF-kB: Macrophages were cultured in 3.5 cm tissue culture dishes at a density of 300,000–400,000 cells per dish. Cells were incubated overnight in an incubator at 37 °C. Macrophages were treated with 30 µM S-nitro blebbistatin (ThermoFisher Scientific, Waltham, MA, USA; #NC0664123) or an equal volume of DMSO (Tocris, Bristol, UK) for 3 h. Cells were then harvested for western blot experiments (see below). Effect of blebbistatin on iNOS induction: Macrophages were pre-treated with 30 uM S-nitro blebbistatin for 4 h and then with 15 µM S-nitro blebbistatin with or without LPS/IFNγ for up to 24 h at 37 °C, 90% humidity and 5% CO_2_ for Western blot experiment and for 4 h at 37 °C, 90% humidity and 5% CO_2_ for ELISA experiments. An equal volume of DMSO was used as a negative control for S-nitro blebbistatin. Western blot and ELISA experimental procedures can be found below.

### 4.5. Western Blot

For Western Blot experiments, cells were cultured overnight in 3.5 cm plastic dishes (CellTreat, Pepperell, MA, USA) before starting treatments. After treatments, cells were washed with cold PBS and harvested by scraping using cell lifters (CellTreat, Pepperell, MA, USA) into 50 µL of RIPA buffer (50 mM Tris-HCl (pH 8) 150 mM NaCl, 0.5% Deoxycholate, 0.1% Sodium Dodecyl Sulfate, 1% NP-40) complemented with 1% protease (ThermoFisher Scientific, Waltham, MA, USA) and phosphatase (Roche, Basel, CH) inhibitors. Cells were centrifuged at 15,000× *g* rpm for 15 min at 4 °C. Supernatants were harvested and the concentration of protein was measured using Precision Red (Cytoskeleton, Denver, CO, USA), with BSA (Jackson Immunoresearch, West Grove, PA, USA) diluted in RIPA buffer used to generate a standard curve. Total protein level and sample volume were equalized and between 8 to 15 µg of protein were run on a Bolt 4–20% Bis-Tris gradient gel (ThermoFisher Scientific, Waltham, MA, USA) at 200 V for 30 min using 1X MES SDS running buffer (Boston BioProducts, Boston, MA, USA) in a mini gel tank (ThermoFisher Scientific, Waltham, MA, USA). After migration, SDS-Page gel was transferred to PVDF membrane (0.45 µm, Thermo Fisher Scientific, Waltham, MA, USA) using the mixed-range molecular weight transfer program (1.3 amps for 7 min) on a Power Blotter semi-dry transfer system (Invitrogen, Waltham, MA, USA). Membrane was blocked for 1 h in 5% non-fat dry milk dissolved in PBS-tween (PBST), followed by primary antibody incubation (with a dilution 1:1000 excepted for GAPDH and iNOS antibodies respectively diluted at 1:20,000 and 1:2000) overnight at 4 °C or 2 h at room temperature in 1% milk in PBS-tween. Primary antibody solutions were washed for 10 min, 3 times with PBST and secondary antibody-HRP (Jackson Immunoresearch, West Grove, PA, USA) solutions were added (dilution 1:10,000 in 5% milk in PBST) for 1 h at room temperature. Membrane was washed 3 times with PBST and chemolumincence was detected using SuperSignal WestPicoPlus (ThermoFisher Scientific, Waltham, MA, USA) and the BioRad Chemidoc Imager. Band intensity was analyzed using ImageJ software. Uncropped blots can be found in Supplemental Figure S7, organized according to the main text figure panel they originate from. When relevant, brightness and contrast changes were applied equally to the entire image and monitored to ensure that signal did not become saturated. Quantification of band intensity was done with ImageJ Software (NIH; 1.53q).

### 4.6. Immunofluorescence Staining

Sterile 12 mm round coverslips (Electron Microscopy Sciences, Hatfield, PA, USA) were coated with Fibronectin (1 µg/mL) (ThermoFisher Scientific; Waltham, MA, USA; #33016015) for 1 h at 37 °C and then washed with sterile PBS. Cells were plated on coverslips at a density of 15,000 cells/well in 24 well plates in fresh macrophage media overnight at 37 °C, 90% humidity and 5% CO2. After treatment with LPS and Interferon gamma, or maintenance in macrophage media, cells were washed with cold PBS and fixed with cold 4% paraformaldehyde (PFA) (for 200 mL solution: 1.69 g NaCl, 0.74 g KCL, 0.035 g CaCl2-2H2O, 0.053 g MgCl2-6H20, 0.033 g NaH2PO4 H2O, 0.364 g glucose, 0.953 g Hepes, 27.36 g M Sucrose, 8 g of PFA and adjust pH at 7.4) for 10 min at RT under a fume hood. Cells were washed three times with PBS and permeabilized with 0.1% Triton X-100 (ThermoFisher Scientific, Waltham, MA, USA) in PBS for 5 min at RT. After three additional washes with PBS, cells were blocked with 5% Bovine Serum Albumin (BSA, Jackson Immunoresearch; West Grove, PA, USA; #001-000-161) and 5% Normal Goat Serum (NGS, Jackson Immunoresearch; West Grove, PA, USA #115-035-068) in PBS for 1 h at RT. Primary antibodies were added (1:75–1:200 in 1% BSA) to the cells for 1 h at RT. After this incubation, primary antibodies were washed three times and incubated with secondary antibodies (ThermoFisher Scientific; Waltham, MA, USA) (1:500 in 1% BSA) and Phalloidin (ThermoFisher Scientific; Waltham, MA, USA) (1:500 in 1% BSA) for 1 h at RT. After this incubation, cells were washed with Hoechst (ThermoFisher Scientific; Waltham, MA, USA) diluted 1:20,000 in PBS for 5 min and then washed 2 more times with PBS. Coverslips were mounted in 15 µL Fluromount G (Electron Microscopy Sciences; Hatfield, PA, USA) on microscopy slides. 

### 4.7. Imaging 

#### 4.7.1. Fixed Cell Imaging

Microscopy slides were imaged at room temperature on an epifluorescence microscope (Olympus IX83; Olympus, Shinjuku City, Tokyo, Japan) connected to an X-cite 120 LED Boost light source (Excelitas Technologies; Waltham, MA, USA for fluorescent imaging. Images were captured using a Hamamatsu digital camera (C13440-20CU). Depending on the experiment, 20× dry or 40×–100× oil immersion objectives were used. Experiments were also conducted on a Zeiss LSM 700 confocal microscope through the USUHS Biomedical Instrumentation Center (BIC) using a dry 20× or 40×–100× oil immersion objectives.

#### 4.7.2. Live Cell Imaging

Cells were plated in a 4 well glass bottom chamber slide (Cellvis; Mountain View, CA, USA) pre-coated with 1 µg/mL fibronectin, at a density of 10,000 cells/well. After 2 h of equilibration, the chamber was placed on the Olympus IX83 microscope in a stable environment at 37 °C, 90% humidity and 5% CO_2_ using the STXG stage top incubator (Tokai Hit Stage top Incubator) connected to an INU incubation system controller (Tokai Hit; Shizuoka-ken, Japan) and imaged for 20 min to 5 h, depending on the experiment. Cells were imaged every 15 s to 5 min, again depending on the experiment, using relief contrast or DIC and/or LED-based fluorescence (X-cite 120 LED Boost, Excilitas Technologies, Waltham, MA, USA with a dry 20×, 40×- or 100× oil immersion objectives. 

#### 4.7.3. Quantitative Image Analysis

Staining intensity and cell area were quantified using FiJi Software (ImageJ 1.52p, NIH USA) to calculate integrated pixel density or object area using the freehand selection tool to outline each cell, or automatically using Cellsens software (Olympus; Shinjuku City, Tokyo, Japan), depending on the experiment. Pearson’s R coefficient was measured using the Coloc2 plugin in Fiji. Circularity factors were obtained by manually drawing a region of interest (ROI) around each individual cell, using phalloidin staining to determine cell edges, and measured by the shape decryption option available through ImageJ (Version 1.52p, NIH USA). Values range from 0 to 1, with cells closer to 1 being defined as more circular. Distance between F-actin peak and nucleus was evaluated using the FiJi Software package (ImageJ 1.52p, NIH USA). Line traces were drawn across the entire cell including the nucleus from left to right. From this solid line, we obtained a plot profile of F-actin and DAPI (nucleus) showing the gray value (fluorescence intensity) as a function of the distance (µm). We then superimposed the F-actin and DAPI plot profiles. We measured the distance (in µm) between the cell’s maximum F-actin fluorescence and its nucleus. Using this same plot profile, we measured the intensity of F-actin fluorescence at the cellular area closest to the nucleus. For the distance of F-actin-nucleus and the F-actin intensity at the nucleus, we obtained 2 values per plot profile (left edge and right edge of the cell). Both the left and right edge values were included in our analysis.

For GFP-NMIIA measures, fluorescence intensities and distance from the leading edge or nucleus was measured in a similar fashion, from solid lines drawn across cells and saved as ROIs. Both the left and right edge values were included in our analysis. Cell Z height was evaluated from confocal z-stacks. The z-step between slices was determined from the image metadata. Sequential images with unique staining were counted, and multiplied by the z-step value to yield an estimate of cell height. 

### 4.8. Elisa

Cells were plated at a density of 225,000 cells/3.5 cm dish for 24 h, then treated with LPS/Interferon gamma containing macrophage media, or macrophage media alone for 24 h. Media was then harvested and stored at −80 °C. IL-6, TNFa and CCL22/MDC1 levels were measured using a mouse IL-6 ELISA kit (Millipore; Burlington, MA, USA; #RAB0308), a mouse Tumor necrosis Factor alpha ELISA kit (Millipore, Burlington, MA, USA; #RAB0477), and mouse CCL22/MDC1 Elisa kit (RayBiotech; Peachtree Corners, GA, USA #ELM-MDC-1). For TNFa, CCL22 and IL6 ELISA measures, supernatants were diluted to 1:20, 1:20 and to 1:1000, respectively, with 1X diluent buffer. The cytokine concentrations were normalized with total protein concentration found in the corresponding dish to correct for cell number.

### 4.9. Chemokine Array

Cells were plated at a density of 225,000 cells/3.5 cm dish overnight, then treated with LPS/IFNγ containing macrophage media, or macrophage media alone for 24 h. Media were then harvested and stored at −80 °C. Media samples were thawed gradually on ice for use in array assay. The manufacturer’s protocol was used to analyze the array (R&D Systems; Minneapolis, MN, USA #ARY020). Briefly, 250 µL of WT or KO LPS/IFNγ-stimulated media was combined with detection antibody cocktail for 1 h and then applied to blocked array membranes. Incubation occurred overnight. Blots were washed the next day, and then HRP-streptavidin was applied for 30 min at room temperature. Arrays were developed using chemiluminescence and imaged on the BioRad Chemidoc Imager. Quantification of band intensity was done using ImageJ software (NIH; 1.53q).

### 4.10. RT-qPCR

Cells were plated with a density of 850,000 cells/10 cm dish for 24 h and were treated with fresh media or LPS/Interferon gamma for 24 h. Cells were washed with cold PBS, scraped in 1 mL of cold PBS, centrifuged at 10,000× *g* rpm for 5 min at 4 °C and pellets were snap frozen and stored in liquid nitrogen. RNA was isolated and purified using RNeasy mini kit (Qiagen; Hilden, Germany #74104). RNA quality and concentration was measured using a Nanodrop (ThermoFisher; Waltham, MA, USA). cDNA were prepared from 600 ng of RNA using Super Script III Reverse Transcriptase (ThermoFisher; Waltham, MA, USA #18080044). qPCR was conducted with iTAQ universal SYBR Green (Biorad; Hercules, CA, USA; #HSS9601), primers described below (IDT; Coralville, IA, USA) and a Biorad CFX96.

TNF-α primers:

5′-TGCCTATGTCTCAGCCTCTTC-3′ and 5′-GGTCTGGGCCATAGAACTGA-3′

IL-6 primers:

5′- TGTGCAATGGCAATTCTGAT-3′ and 5′-GGTACTCCAGAAGACCAGAGGA-3′

Nos2 primers:

5′-GCTCATGACATCGACCAGAA-3′ and 5′-TGTTGCATTGGAAGTGAAGC-3′

GAPDH primers:

5′-TCTCCACACCTATGGTGCAA-3′ and 5′-CAAGAAACAGGGGAGCTGAG-3′

The PCR program used was 30 s at 98 °C, (10 s at 98 °C, 30 s at 60 °C) for 40 cycles, 65 °C to 95 °C with 0.5 °C increment 2 s/step. The expression fold change of a gene was calculated with 2^−ΔΔCt^.

### 4.11. Nitrite Concentration Measurement

Nitrite concentration was measured using the Nitrite Colorimetric Assay Kit (Cayman Chemicals; Ann Arbor, MI, USA; #780001). The addition of Griess Reagents converts nitrite (produced by nitric oxide accumulation in media) into a purple azo compound. The absorbance at 540 nm of duplicate samples was then measured using a CLARIOstar plate reader and converted into the nitrite concentration (µM) using a standard curve also performed in duplicate. This concentration is then corrected for background media nitrite levels using the macrophage media as a blank. Lastly, each sample measurement is normalized relative to cell density using representative cell counts collected immediately prior to harvesting the supernatant.

### 4.12. Proteasome Inhibitor Treatment

Macrophages were cultured in 3.5 cm tissue culture dishes at a density of 300,000–400,000 cells per dish. Cells were incubated overnight in a tissue culture incubator prior to treatment. Four conditions were utilized: (1) LPS (500 ng/mL) and IFNγ (25 pg/mL) for 26 h; (2) LPS (500 ng/mL), IFNγ (25 pg/mL) and blebbistatin (30 µM) for 26 h; (3) LPS (500 ng/mL), IFNγ (25 pg/mL) and blebbistatin (30 µM) for 26 h with 10 µM MG132 added for the final 2 h (LPS, IFNγ and blebbistatin were present during the MG132 treatment); (4) control group, consisting of DMSO applied at an equal volume as blebbistatin. The experiment was ended with three washes with cold PBS and RIPA lysates collected and processed as outlined above.

### 4.13. FACS Analysis

Wild type and *Arpc2-/-* macrophages were harvested by incubation with 0.5 mM EDTA at 4 °C for 10 min, followed by scraping into FACS buffer (2% FBS in DPBS). Cells were collected by centrifugation at 400× *g* for 5 min, and the supernatant was aspirated. Fc receptors were blocked with the Fc Blocker Reagent (Innovex Biosciences; Richmond, CA, USA; #NB335) for 30 min on ice. Cells were then washed with FACS buffer and centrifuged twice, as above. Cells were incubated with APC-conjugated MHCII antibody on ice for 30 min in the dark. Cells were then washed with FACS buffer and centrifuged twice, as above. After the final wash cells were resuspended in FACS buffer containing EDTA to prevent cell clumping and taken for FACS analysis. Ten events WT or *Arpc2-/-* macrophages were sorted for each condition (±500 ng/mL LPS and 25 pg/mL IFNγ) on a BD LSRII Flow Cytometer Cell Analyzer (BD Biosciences; Franklin Lakes, NJ, USA) at the USUHS Biomedical Instrumentation Center. Gates were set to select singlets, and then to separate cells from debris. SYTOX™ Blue (ThermoFisher Scientific; Waltham, MA, USA; #S34857) was used to differentiate dead cells from live. APC fluorescence was recorded for 10,000 events for each condition, and only live cells were used for quantification. Results were plotted with FlowJo (FlowJo, LLC.; Stanford University, Palo Alto, CA, USA) and median APC fluorescence intensity was reported.

### 4.14. Statistics

All statistics were calculated from the average of independent experimental means using GraphPad Prism (Prism 7 version 7.05; San Diego, CA, USA). The *p* values and statistical tests utilized are indicated directly on the figures and legends.

## Data Availability

All primary data will be openly available at an online repository prior to publication.

## Supplementary Materials

The following supporting information can be downloaded at: https://www.mdpi.com/article/10.3390/cells11111806/s1, Figure S1: Height of WT macrophages at baseline and after 20 min of LPS/IFNγ stimulation; Figure S2: GFP-myosin IIA is dynamically reorganized to the nucleus and cell edge during the acute phase of LPS/IFNγ stimulation; Figure S3: Arpc2-/- macrophage characterization; Figure S4: Total levels of p65, Erk1/2 and p38 after Arp2/3 complex or myosin II disruption; Figure S5: NF-kB translocation and inflammation-associated transcripts are unaffected by cytoskeletal perturbations; Figure S6: Myosin II inhibition by blebbistatin does not impact IL6 or TNFa secretion; Figure S7: Uncropped Western blot images; Movie S1: WT macrophages responding to 500 ng/mL LPS + 25 pg/mL IFNγ wash-in; Movie S2: WT macrophages after mock wash-in of normal macrophage media.
