# Supplementary material for: The Actin Cytoskeleton Responds to Inflammatory Cues and Alters Macrophage Activation
Source: Cells. 2022 May 31;11(11):1806. doi: 10.3390/cells11111806 (PMC9180445; doi:10.3390/cells11111806)
Supplement: Supplementary file 1 [file cells-11-01806-s001.zip › Supplementary materials_finalv2.pdf]

SUPPLEMENTARY DATA

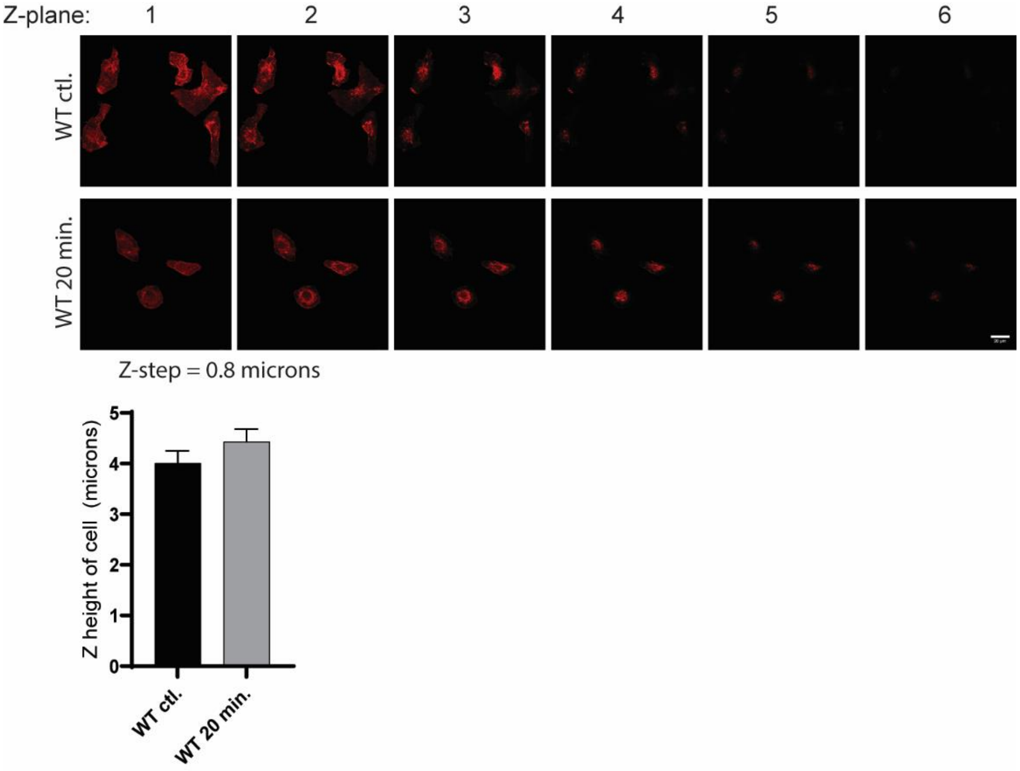

Supplementary Figure S1: Height of WT macrophages at baseline and after 20 minutes of LPS/IFNγ stimulation. *Top*, confocal z-slices of phalloidin staining in PFA-fixed cells from bottom (z-plane 1) to top of the cell (z-plane 6). *Bottom*, Average Z height of WT macrophages at baseline and after 20 minutes of LPS/IFNγ stimulation, in microns. Z-step between stack images is 0.8 microns. Scale bar = 20 microns. N = 3 experiments, with at least 11 cells imaged per condition in each experiment. Error bars represent standard error of the mean.

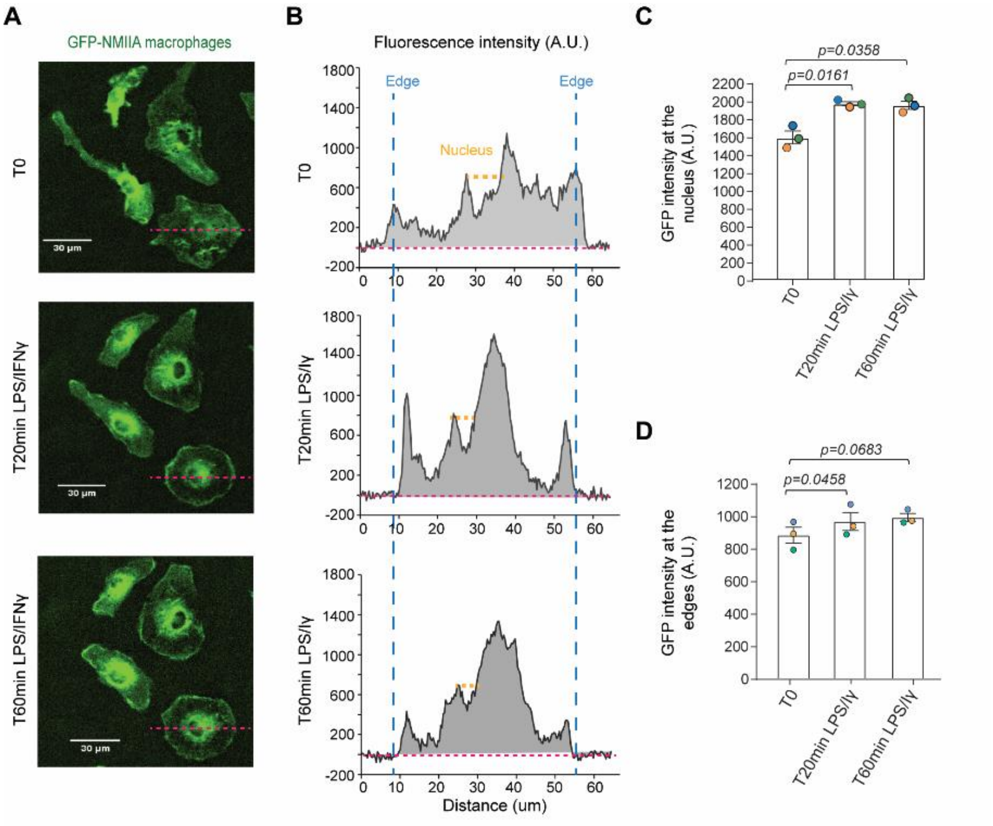

Supplemental figure S2: GFP-myosin IIA is dynamically reorganized to the nucleus and cell edge during the acute phase of LPS/IFNγ stimulation. A) Representative time lapse imaging of GFP-NMIIA macrophages at T0, T20min and T60min of stimulation treatment. Scale bar = 30 µm. Dashed line illustrates representative trace of GFP-myosin IIA used to generate quantitative data derived from this experiment. B) Fluorescence intensity spectrums from a cross section (pink dashed line in panel A) at T0, T20min and T60min of stimulation treatment. Nuclear diameter is noted by horizontal yellow line marking areas of low GFP signal corresponding to the line trace passing over the nucleus. The original cell edge is noted with vertical blue line. C) Average of GFP intensity, as measured by line trace pixel intensity values, at the nucleus and D) at the edge of the cells, n= 3 independent fields of view, with more than 20 cells per frame, p values were obtained with a paired t-test and $p \leq 0.05$ was considered significant. Individual experimental means are color coded, and error bars represent standard error of the mean.

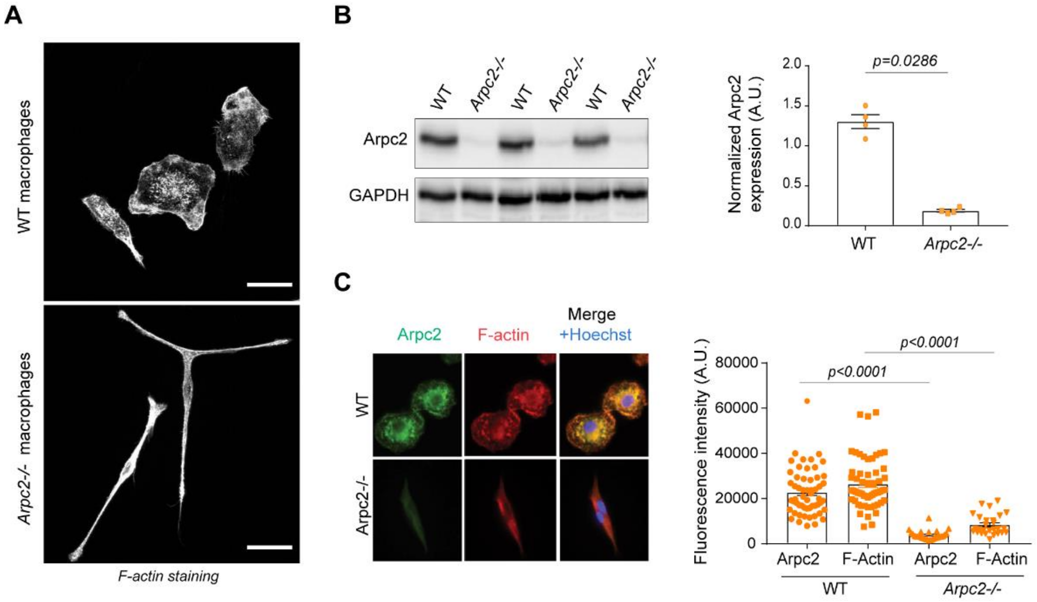

Supplemental Figure S3: *Arpc2-/-* macrophage characterization. A) Representative Z stack confocal images of WT (top) and *Arpc2-/-* (bottom) macrophages stained with Phalloidin (F-actin staining). Scale bar = 30 µm. B) Left: Representative immunoblot of Arpc2 and GAPDH from WT and *Arpc2-/-* RIPA protein lysates. Right: Quantification of Arpc2 expression, normalized to GAPDH, in WT and *Arpc2-/-* protein lysates, n=3 independent experiments. P values were obtained with an unpaired t-test, with $p \leq 0.05$ considered significant. Error bars represent standard error of the mean. C) Left*:* Representative epifluorescence images of WT and *Arpc2-/-* macrophages after being immuno-stained for arpc2 and Phalloidin (F-actin). Right: Quantification of fluorescence intensity for Arpc2 and F-actin from WT and *Arpc2-/-* macrophages, n = at least 25 cells per genotype. P values were obtained with an unpaired t-test, with $p \leq 0.05$ considered significant. Error bars represent standard error of the mean.

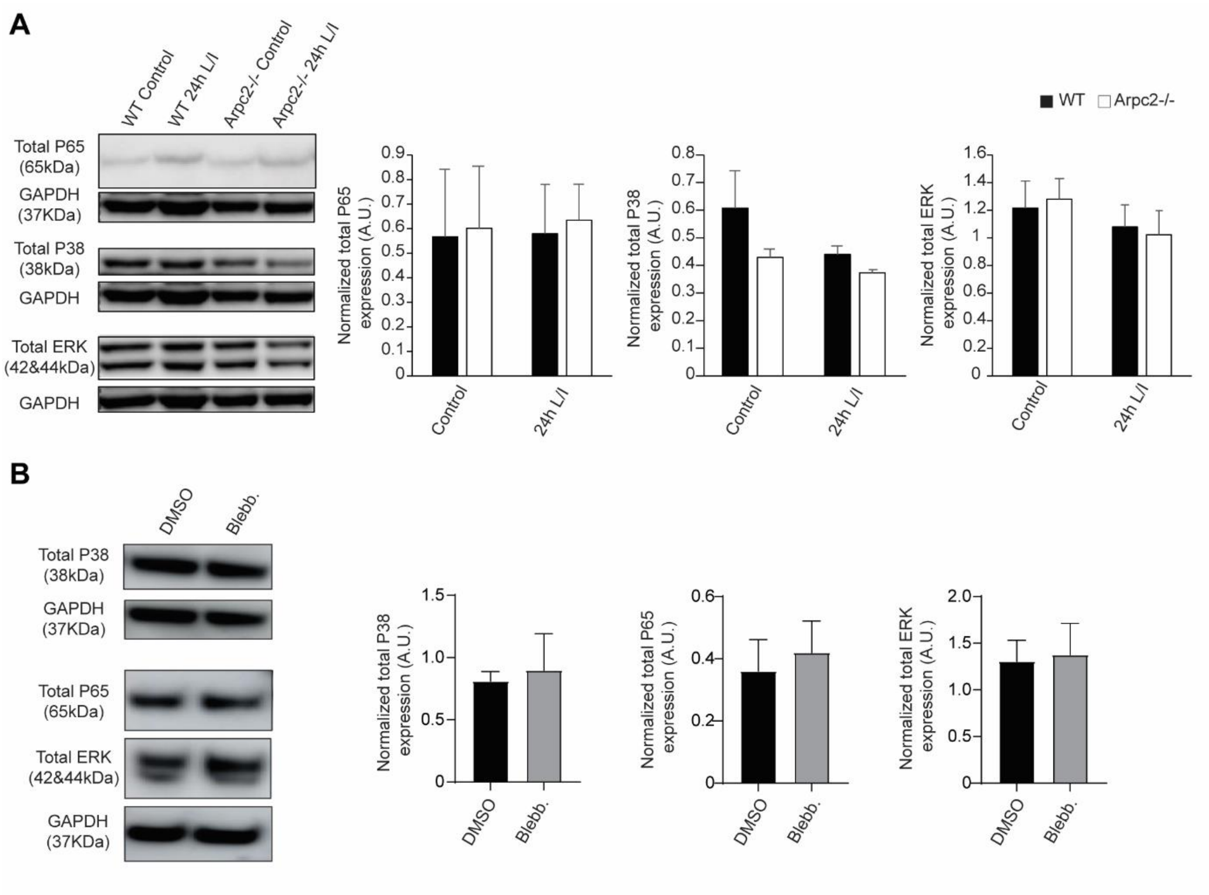

Supplemental Figure S4: Total levels of p65, Erk1/2 and p38 after Arp2/3 complex or myosin II disruption. A) *Left*, representative immunoblots of total p65, p38, total Erk1/2 and GAPDH, from unstimulated WT and *Arpc2-/-* RIPA-extracted protein lysates at baseline and after 24h LPS/IFNγ stimulation. *Right*, average chemiluminescence level (normalized to GAPDH) from N = 3 independent experiments. Error bars represent standard error of the mean. B) *Left*, representative immunoblots of total p38, total p65, total Erk1/2 and GAPDH, from unstimulated WT macrophages treated with either DMSO- or 30 µM blebbistatin for 2 hours. Proteins were extracted with RIPA buffer. *Right*, average chemiluminescence level (normalized to GAPDH) from N = 4 independent experiments for Erk1/2 and p65, and N = 3 independent experiments for p38. Error bars represent standard error of the mean. Molecular weights of all proteins are indicated alongside the representative blot.

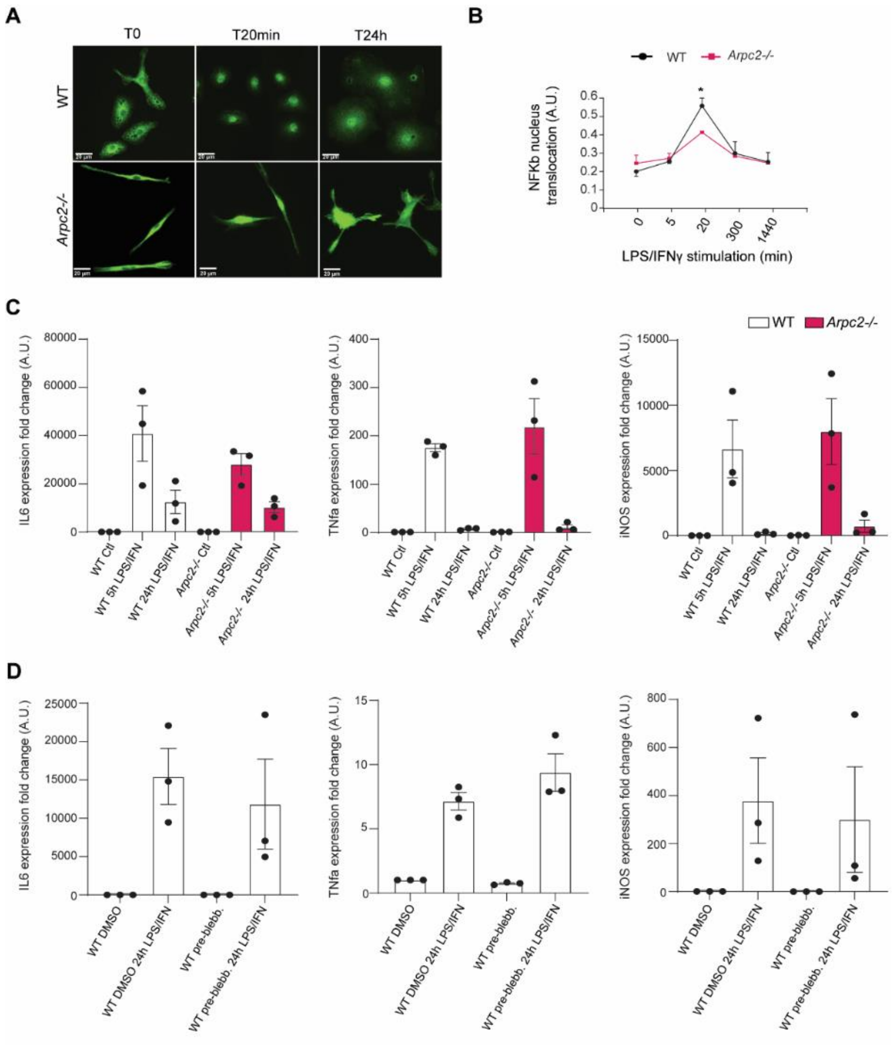

Supplemental Figure S5: NF-kB translocation and inflammation-associated transcripts are unaffected by cytoskeletal perturbations. A) Representative epifluorescence images of the p65 subunit of NF-kB imaged via indirect immunofluorescence in WT and *Arpc2-/-* macrophages under control conditions, after 20min or after 24h of LPS/IFNγ stimulation. Scale bar = 20 microns. B) Average NF-kB nuclear translocation in WT and *Arpc2-/-* macrophages under control conditions, after 20 min or after 24h of LPS/IFNγ stimulation from 2 independent experiments. Data represents quantification of nuclear p65 signal. P values were obtained with an unpaired t-test, with *p$\leq$ 0.05 considered significant. Error bars represent standard error of the mean. C) WT or *Arpc2-/-* macrophage induction of IL6, TNFα and iNOS genes analyzed via qPCR and expressed as fold change above WT unstimulated samples. RNA was isolated from unstimulated cells or cells activated for 5 or 24h with 500 ng/mL LPS + 25 pg/mL IFN, data is from 3 independent experiments. Error bars represent standard error of the mean. D) WT macrophages were pre-treated with DMSO or 30 µM blebbistatin, followed by stimulation with LPS/IFNγ in the presence of 15 µM blebbistatin. Induction of IL6, TNFα and iNOS genes was assayed via qPCR and expressed as fold change above DMSO unstimulated samples. RNA was isolated from unstimulated cells or cells activated for 24h with 500 ng/mL LPS + 25 pg/mL IFN, data is from 3 independent experiments. Error bars represent standard error of the mean.

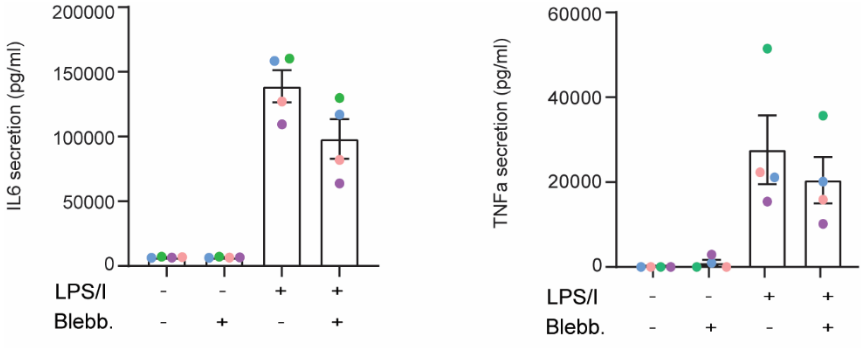

Supplemental Figure S6: **Myosin II inhibition by blebbistatin does not impact IL6 or TNFa secretion.** Average of IL6 and TNFα cytokine secretion for WT macrophages left untreated or stimulated with 500 ng/mL LPS + 25 pg/mL IFNγ ± 15 µM blebbistatin, from 4 independent experiments. Cells were pre-treated with 30 µM blebbistatin for 4h followed by treatment with LPS/IFNγ and 15 µM blebbistatin, as indicated above. Individual experimental means are color coded and error bars represent standard error of the mean. Unpaired t-tests were used to analyze significance, and no significant p-values were detected.

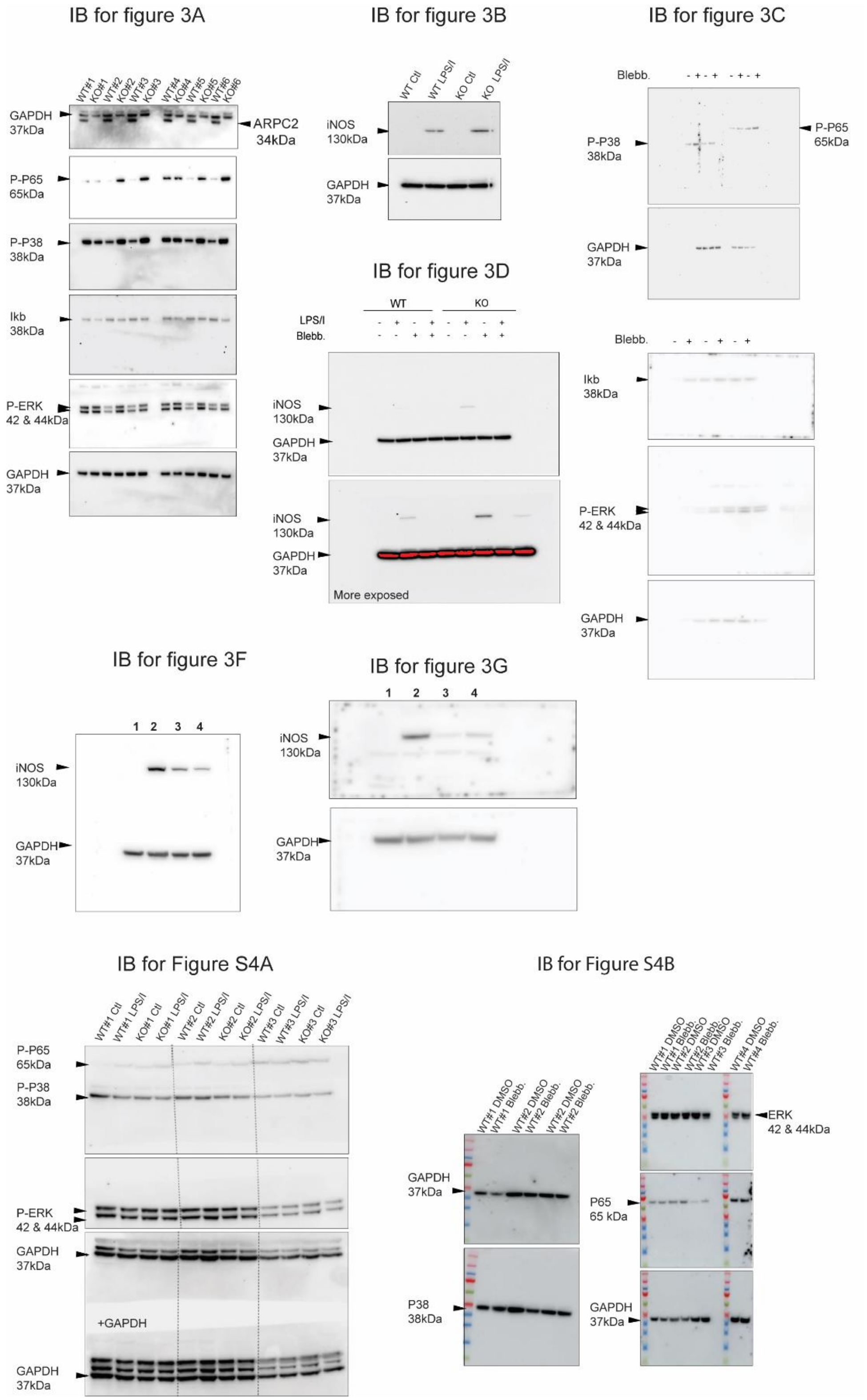

Supplemental Figure S7: Uncropped western blot images. Notes: 1) For Figure 3A – p-p65, p-p38 and Arpc2 were all probed from the same membrane, with GAPDH loading control. P-Erk and IkB were on a

different membrane, with a different GAPDH blot as loading control. Multiple biological replicates were run on the same gel, as indicated. 2) For Figure 3C – p-p38 and p-p65 were blotted on the same membrane from the same biological samples, separated by a central protein ladder lane. GAPDH accompanies these blots. P-Erk and IkB were blotted on a second membrane, with another GAPDH blot used to quantify loading. Multiple biological replicates were loaded on the same gels, as indicated. 3) Figure 3F, 3G: Numbers correspond to treatment conditions that can be found in schematic form in the corresponding main text figure. All blots in this SF are uncropped, but boxes have been placed around the uncropped blots to differentiate them from one another and the white page background. 4) Figure S4A: All three experimental N were run on the same blot. Figure S4B: All three (for p38) or four (for p65 and Erk ½) experimental N were run on the same blot. Blots are grouped together and chemiluminescence was merged with the color image showing the molecular weight protein ladder.
